# Supplementary figures and images for: Melatonin attenuates MPP+-induced autophagy via heat shock protein in the Parkinson’s disease mouse model
Source: PeerJ. 2025 Jan 21;13:e18788. doi: 10.7717/peerj.18788 (PMC11758912; doi:10.7717/peerj.18788)

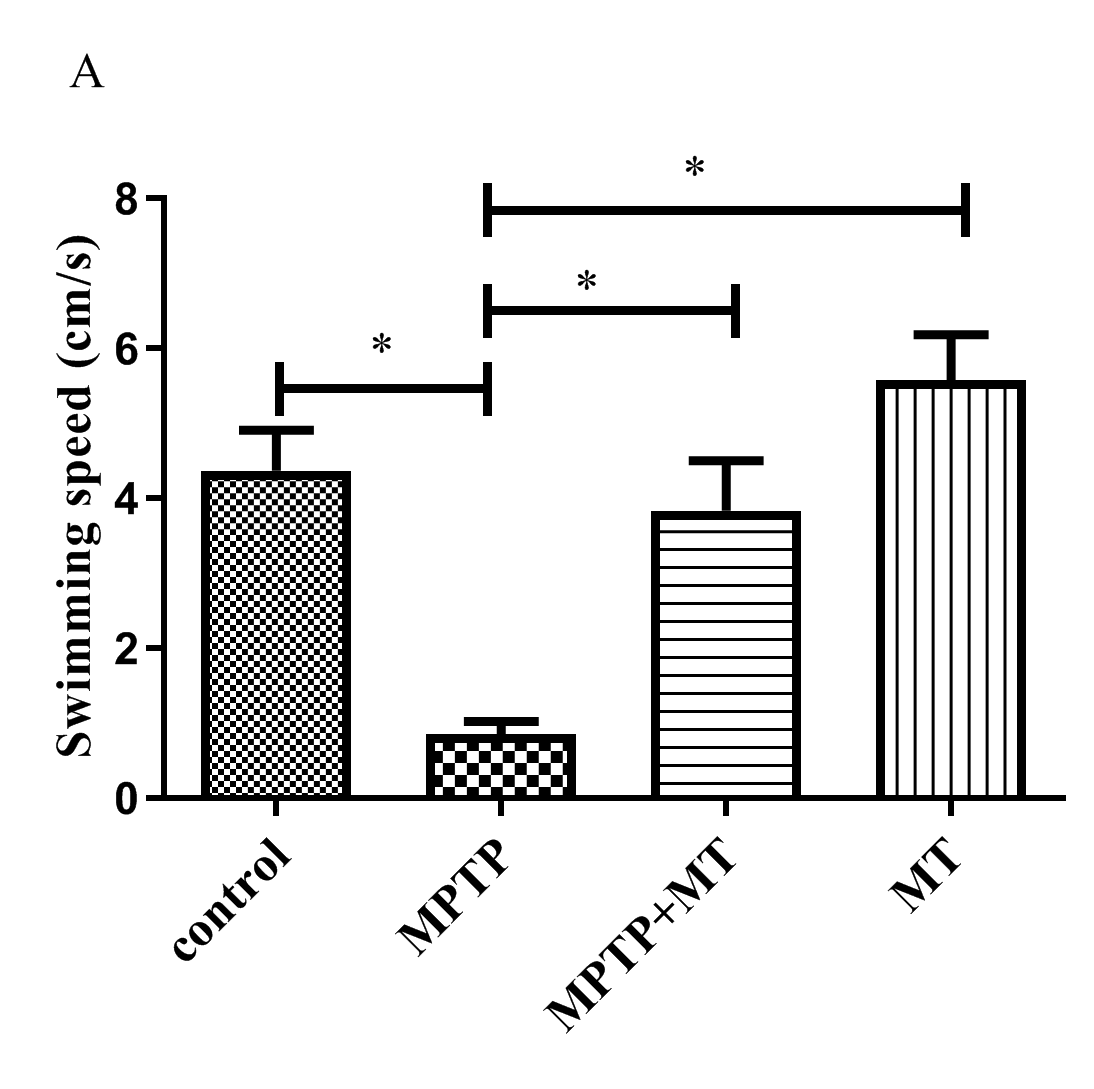

Supplement: Supplemental Information 1 [file peerj-13-18788-s001.zip › Supplemental/Figrue 1B.png]

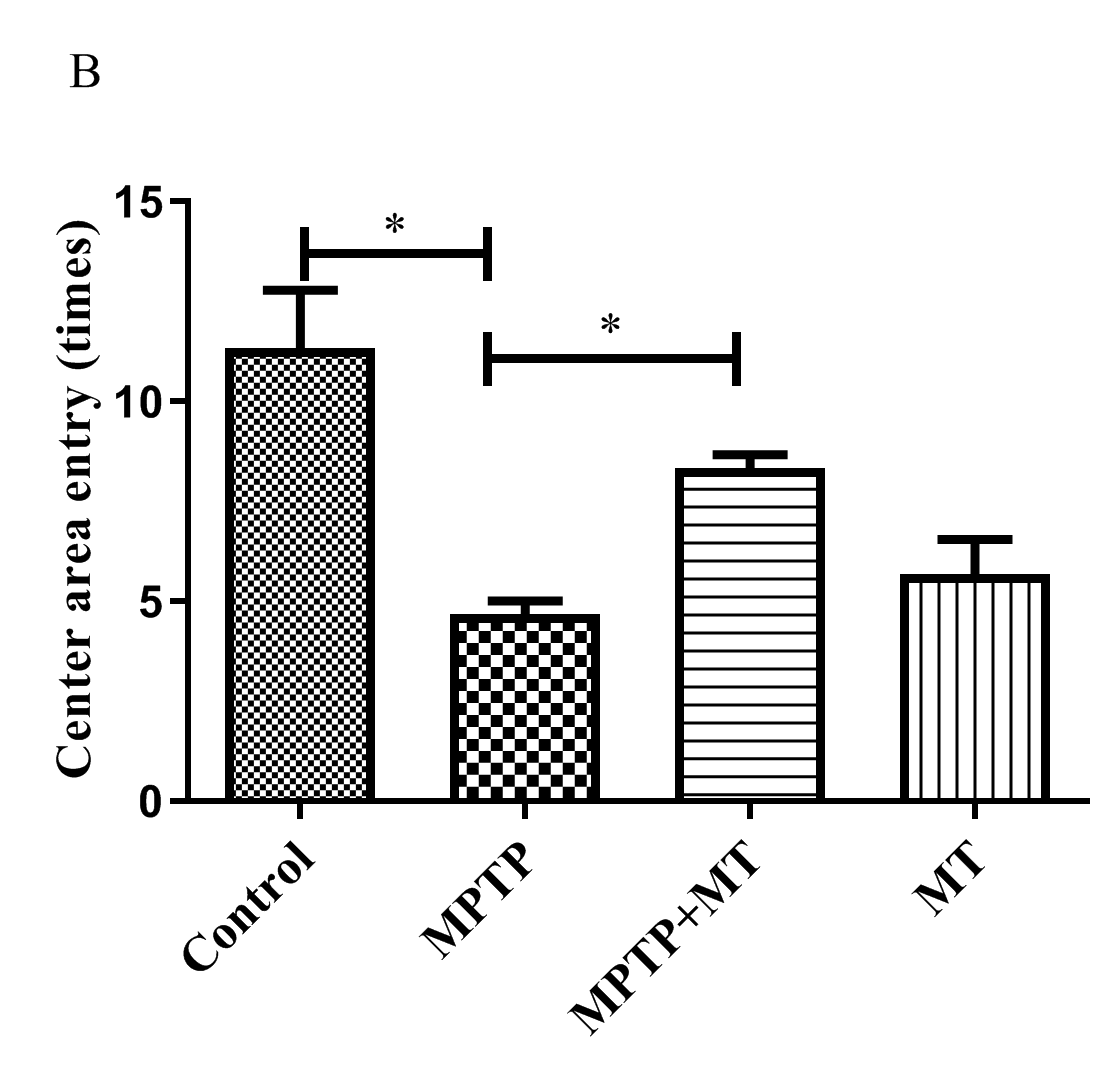

Supplement: Supplemental Information 1 [file peerj-13-18788-s001.zip › Supplemental/Figrue 1C.png]

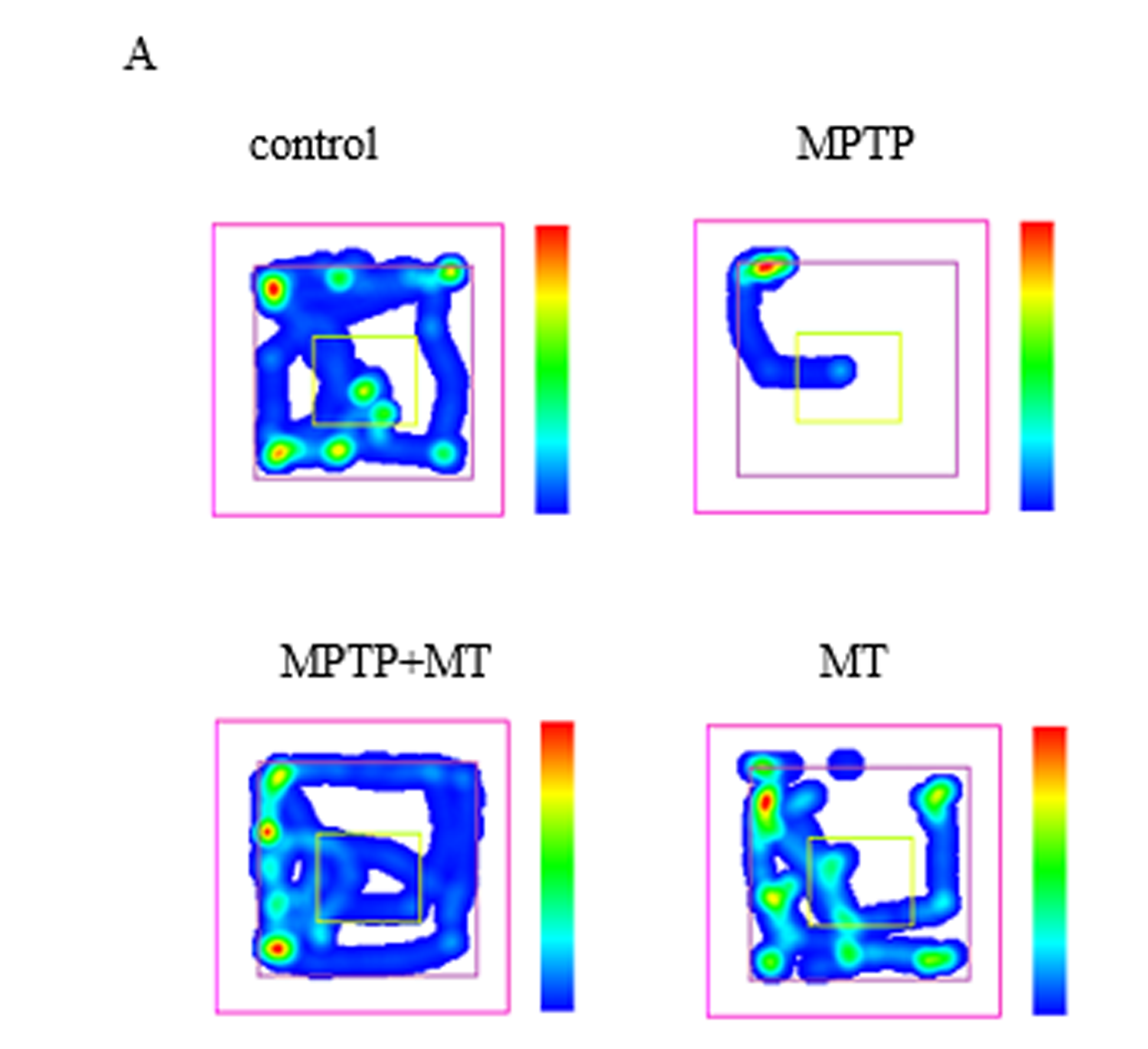

Supplement: Supplemental Information 1 [file peerj-13-18788-s001.zip › Supplemental/Figure 1A.png]

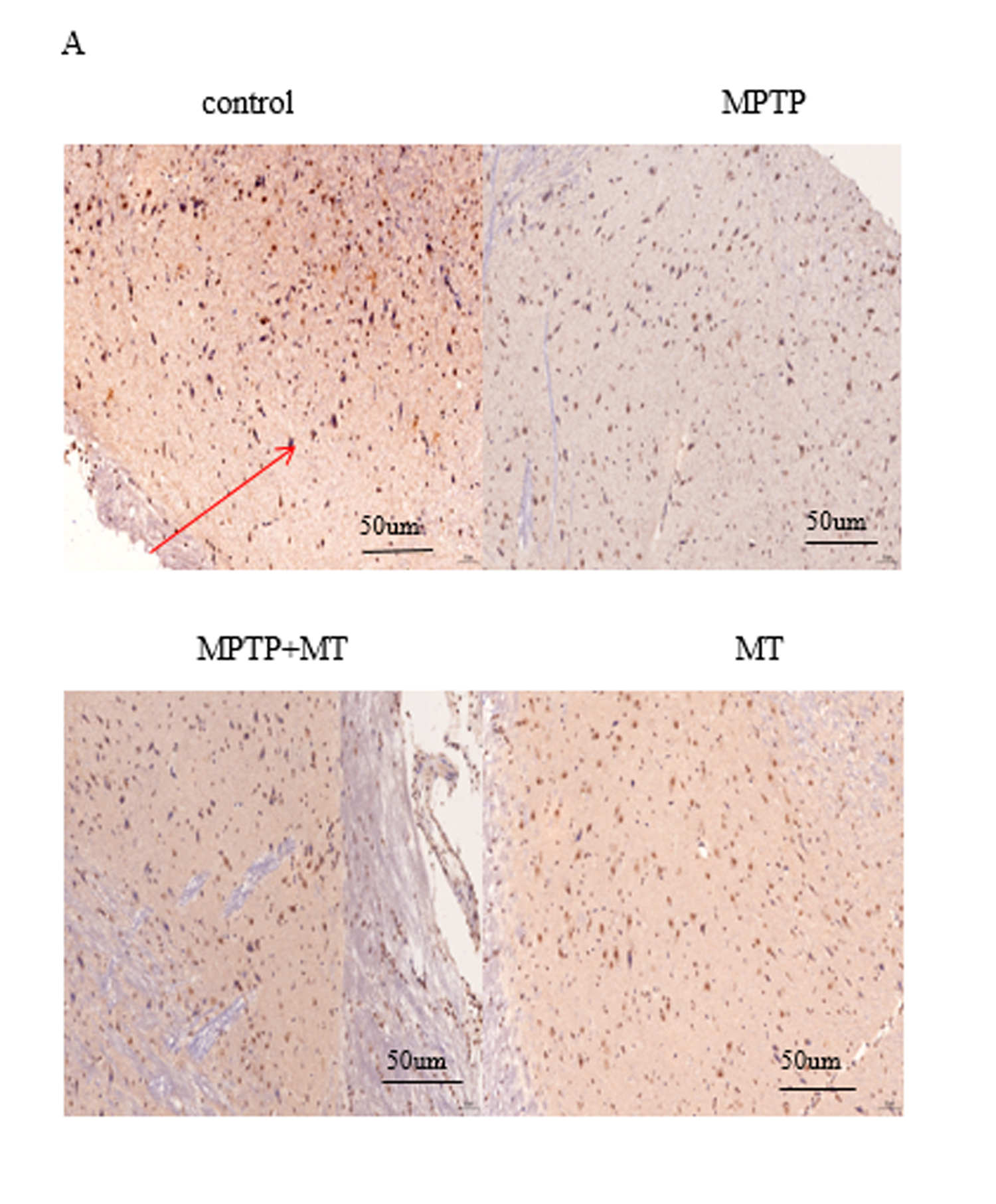

Supplement: Supplemental Information 1 [file peerj-13-18788-s001.zip › Supplemental/Figure 2A.png]

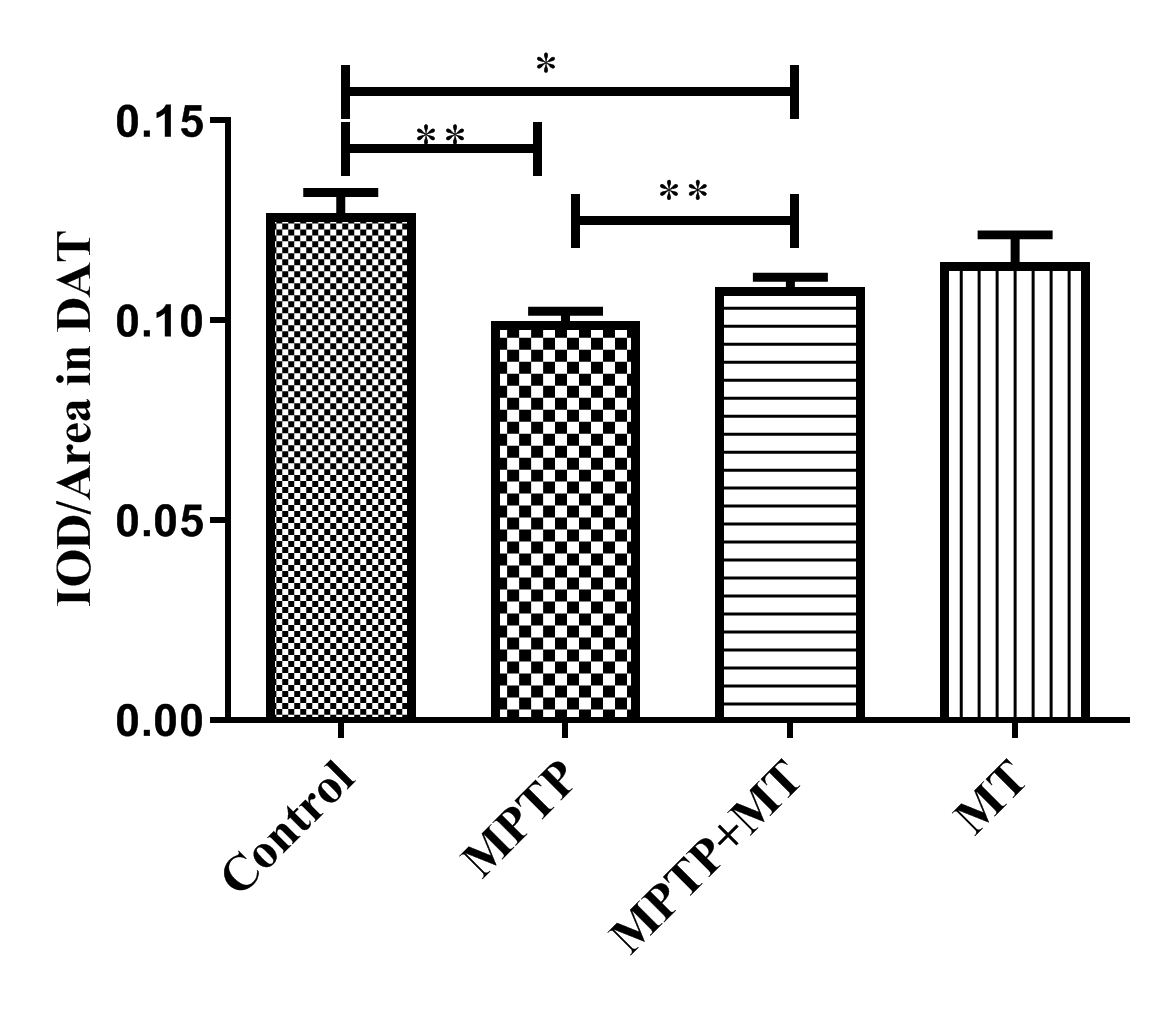

Supplement: Supplemental Information 1 [file peerj-13-18788-s001.zip › Supplemental/Figure 2B.png]

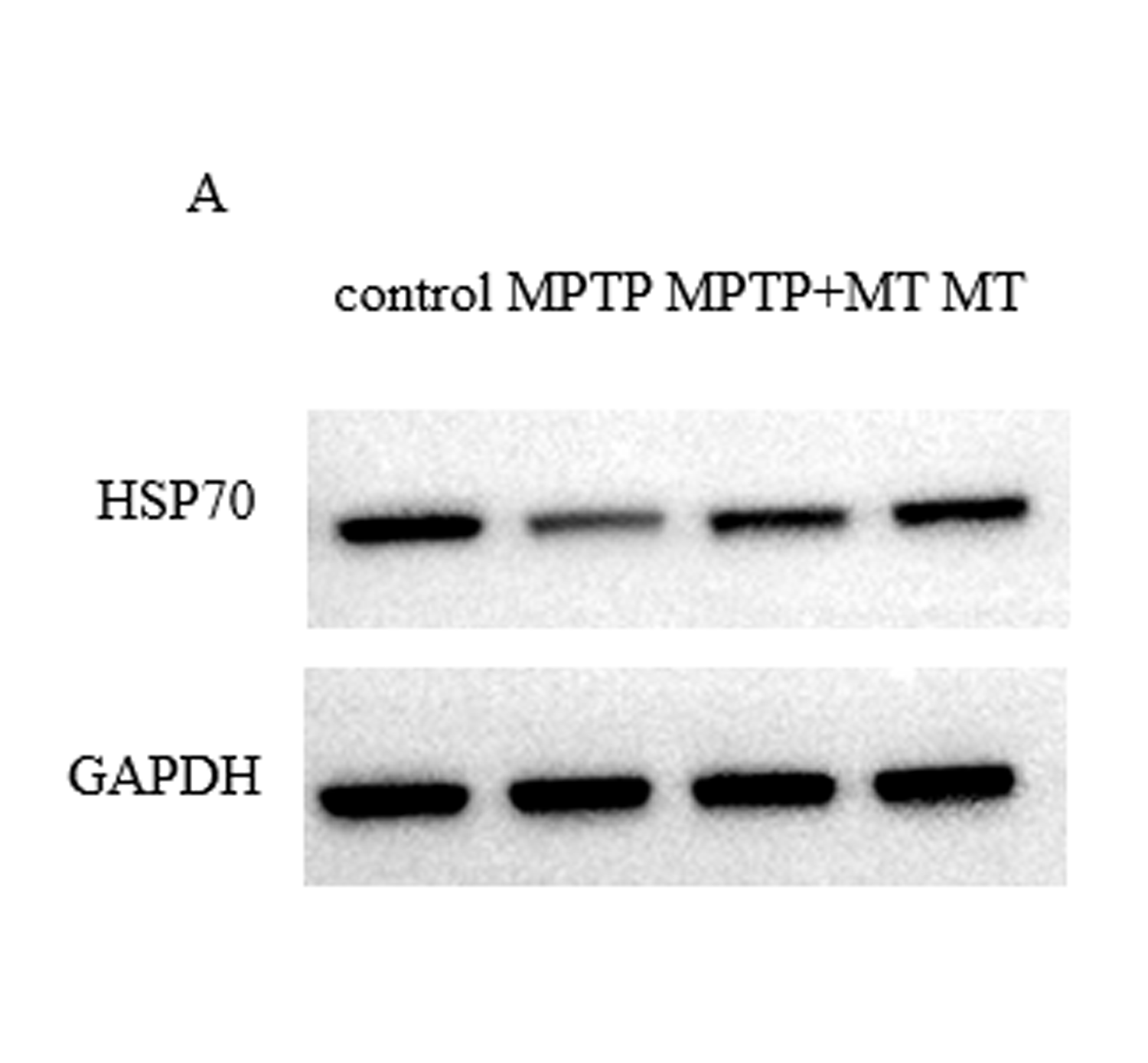

Supplement: Supplemental Information 1 [file peerj-13-18788-s001.zip › Supplemental/Figure 3A.png]

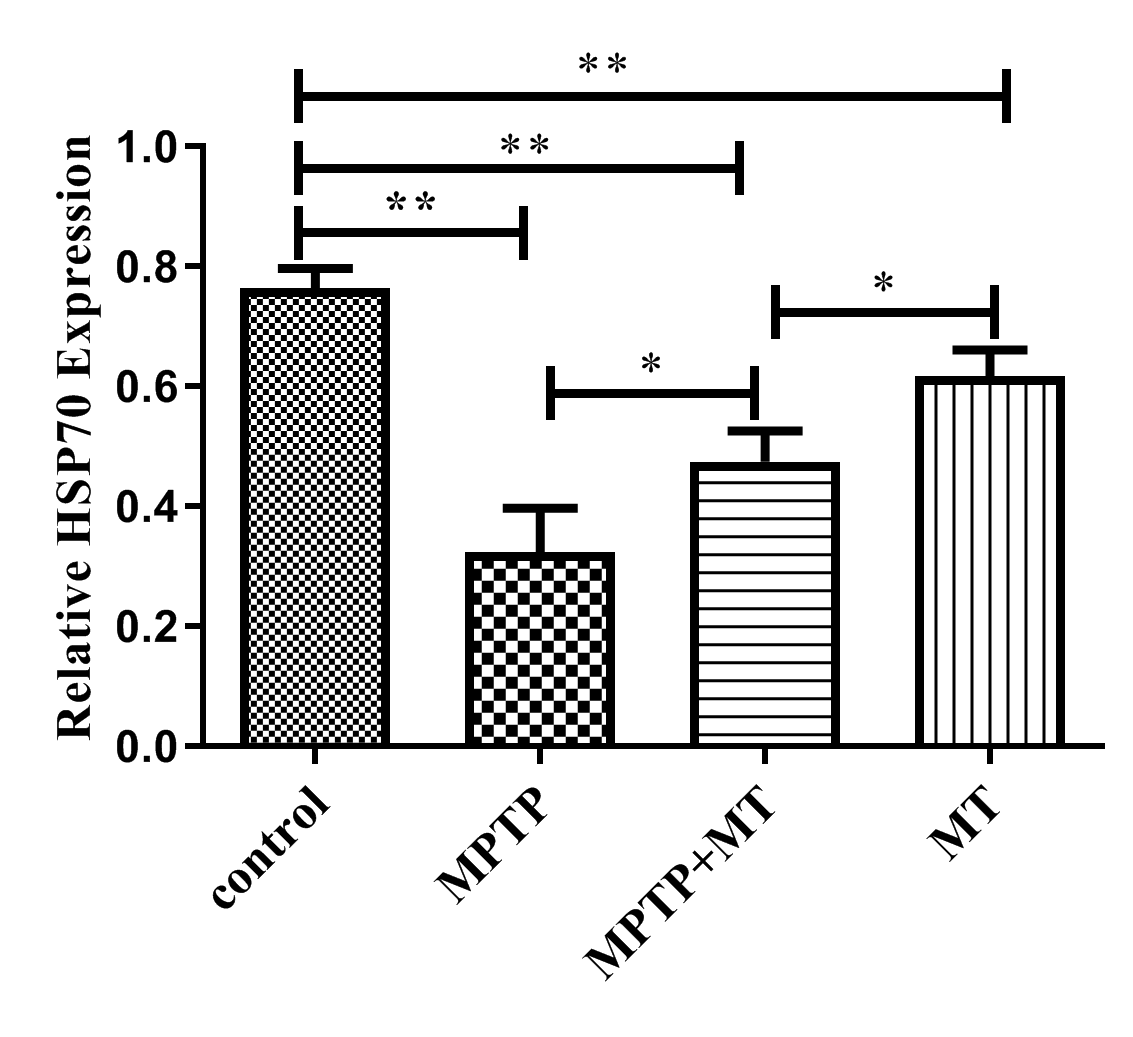

Supplement: Supplemental Information 1 [file peerj-13-18788-s001.zip › Supplemental/Figure 3B.png]

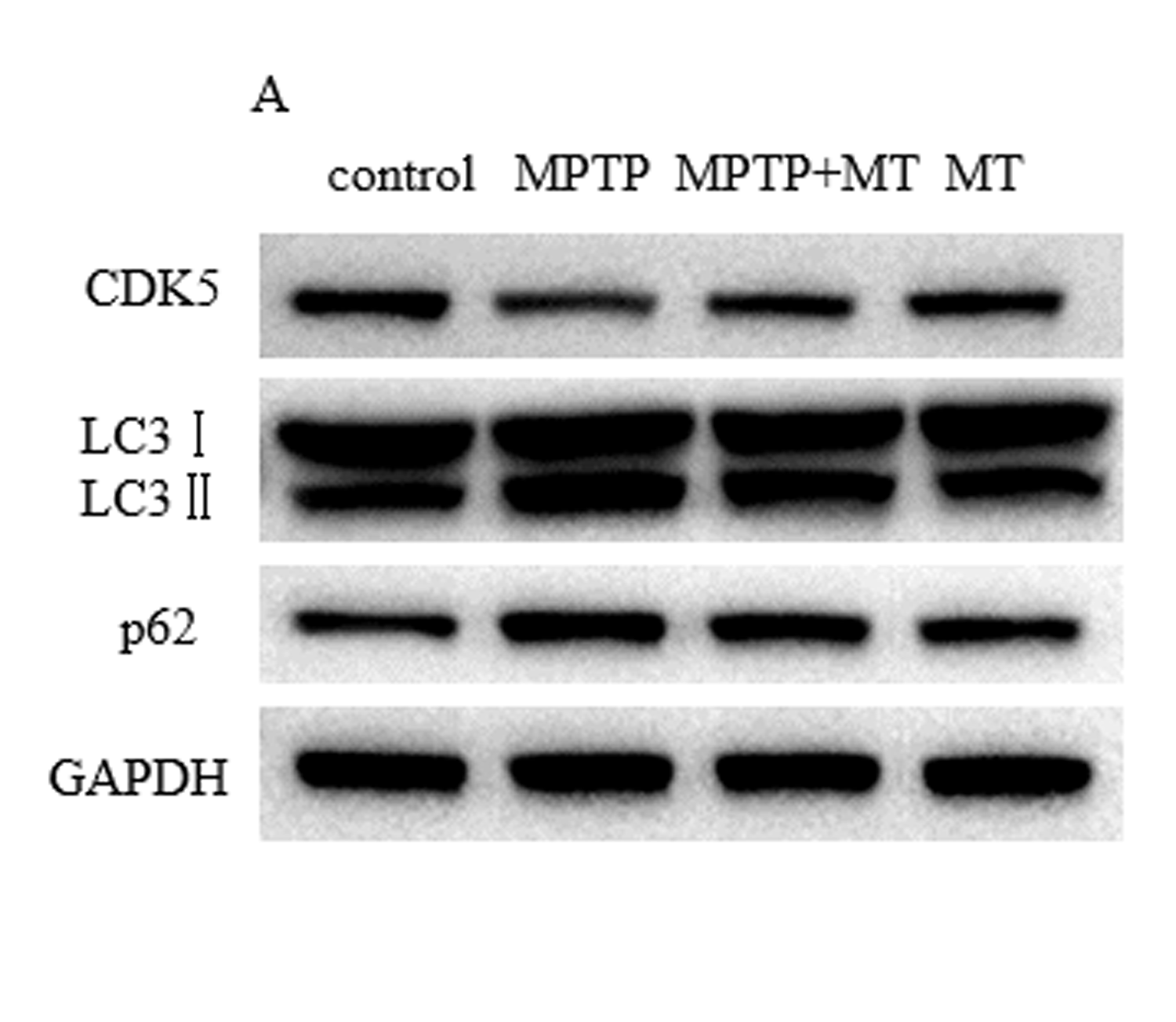

Supplement: Supplemental Information 1 [file peerj-13-18788-s001.zip › Supplemental/Figure 4A.png]

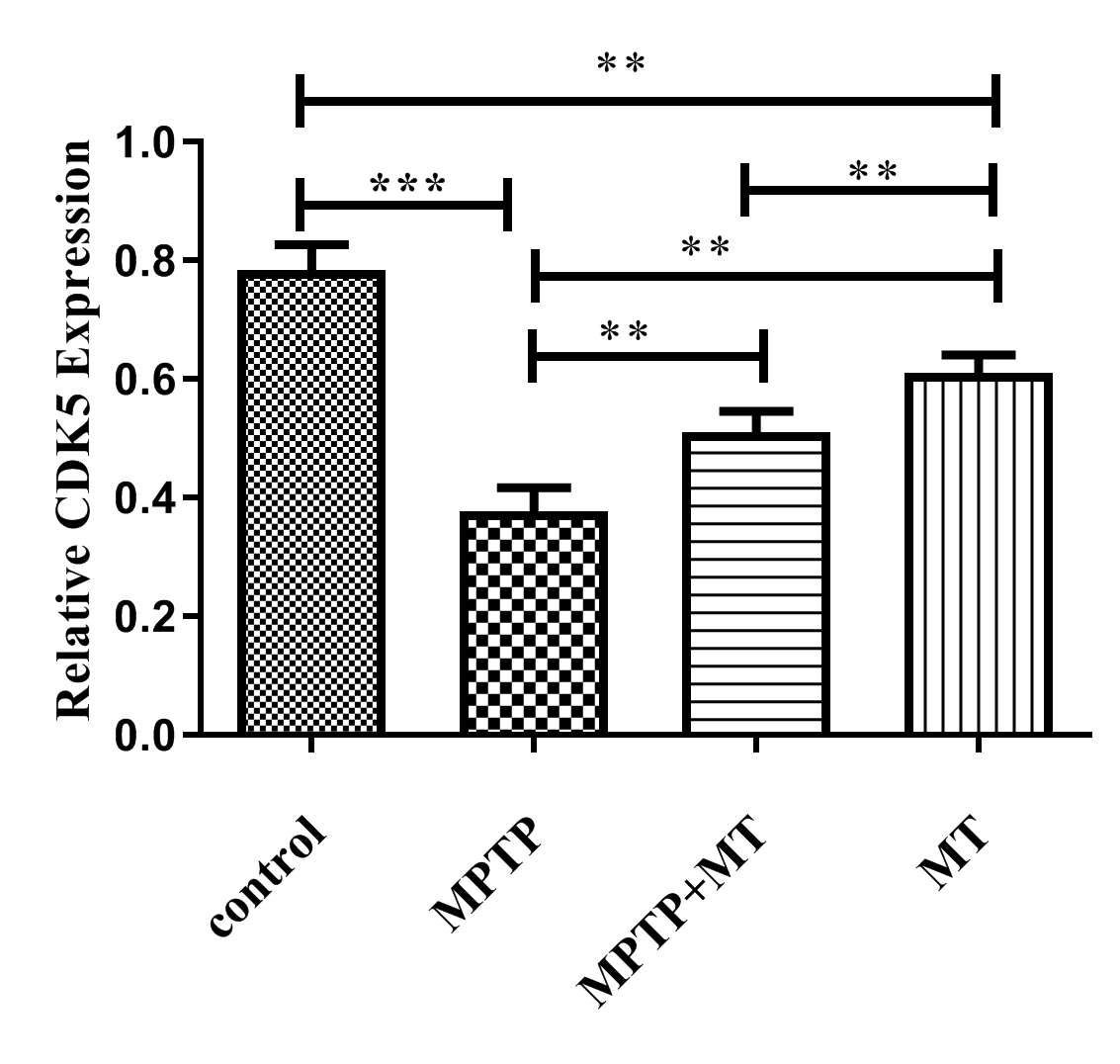

Supplement: Supplemental Information 1 [file peerj-13-18788-s001.zip › Supplemental/Figure 4B.png]

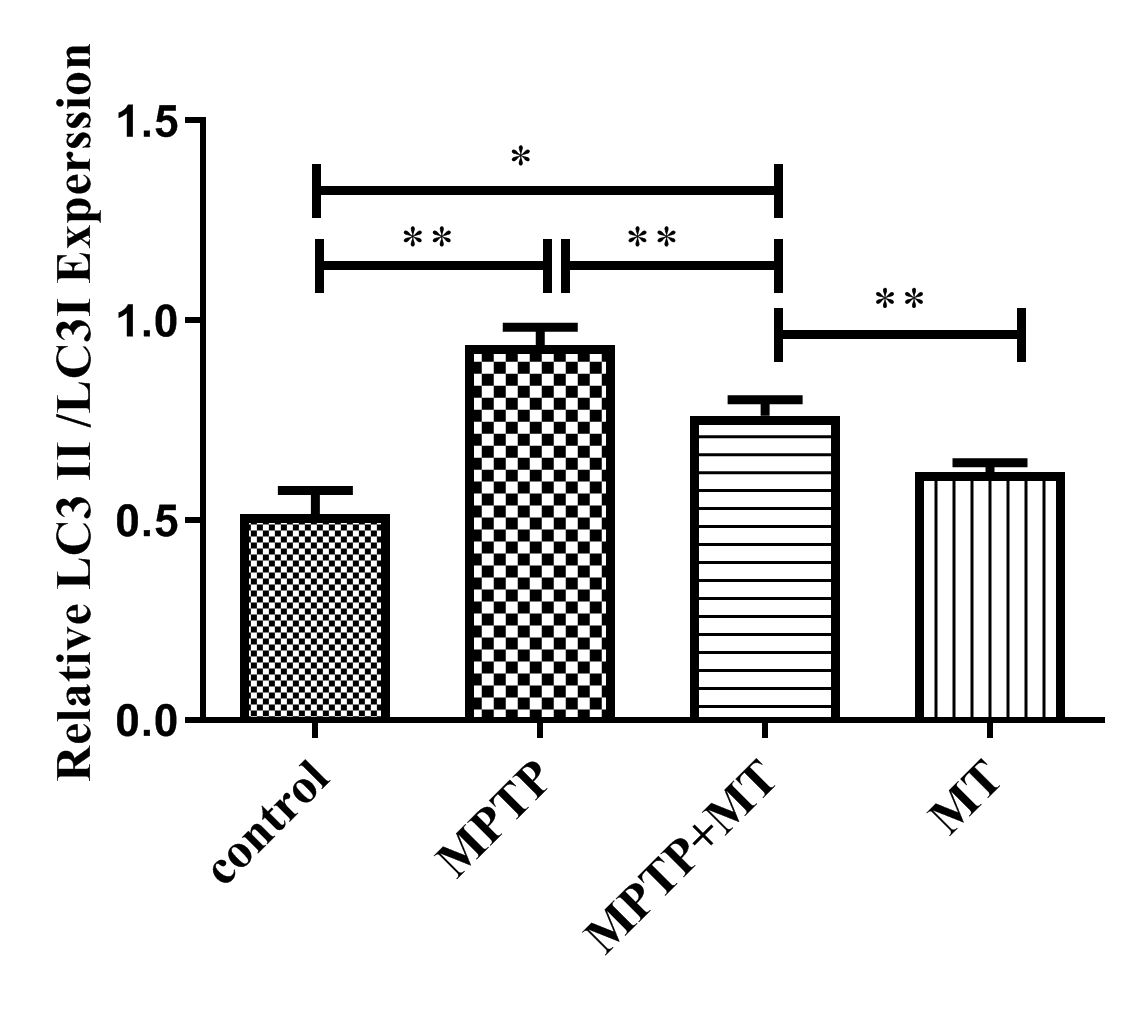

Supplement: Supplemental Information 1 [file peerj-13-18788-s001.zip › Supplemental/Figure 4C.png]

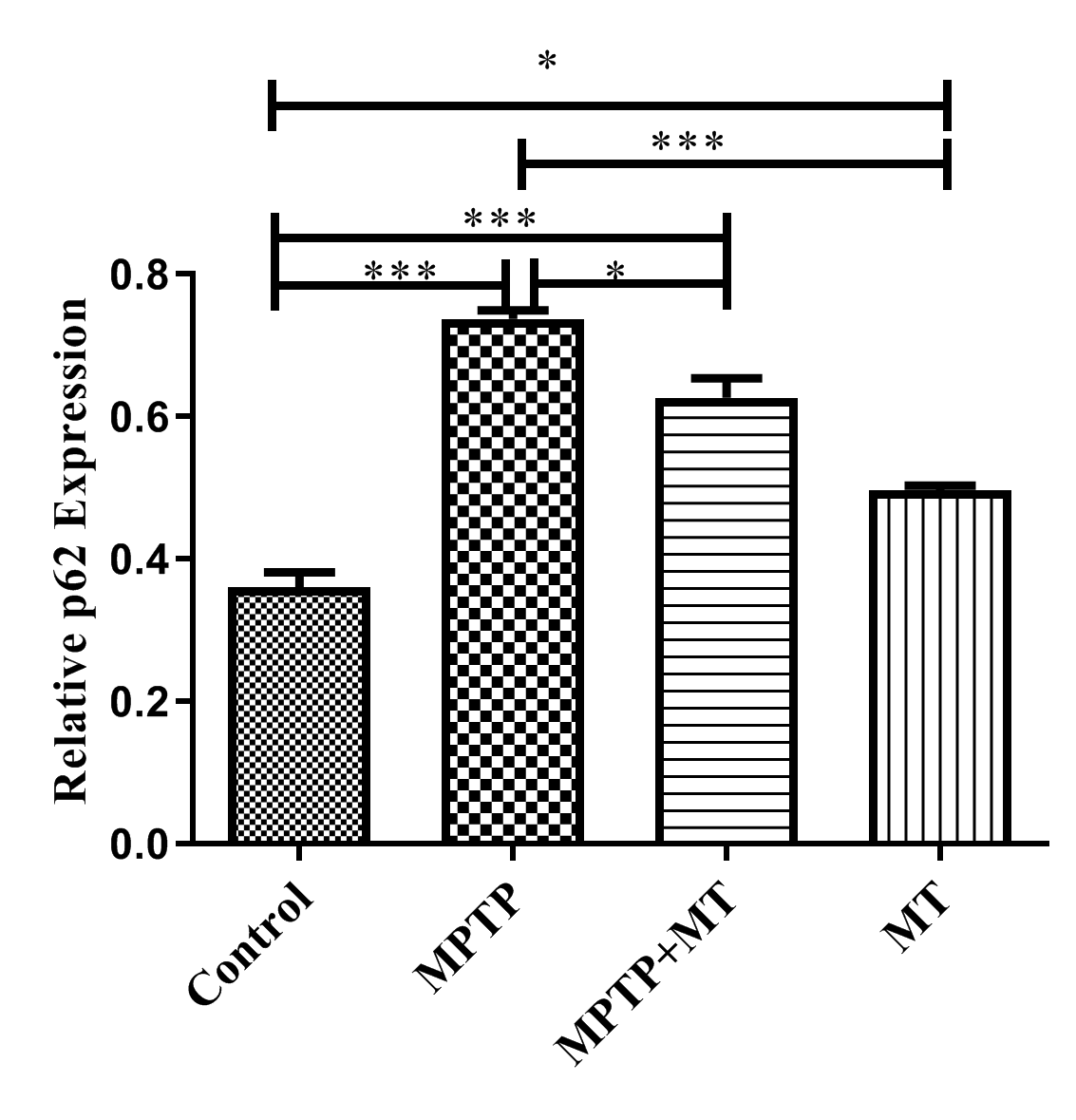

Supplement: Supplemental Information 1 [file peerj-13-18788-s001.zip › Supplemental/Figure 4D.png]
